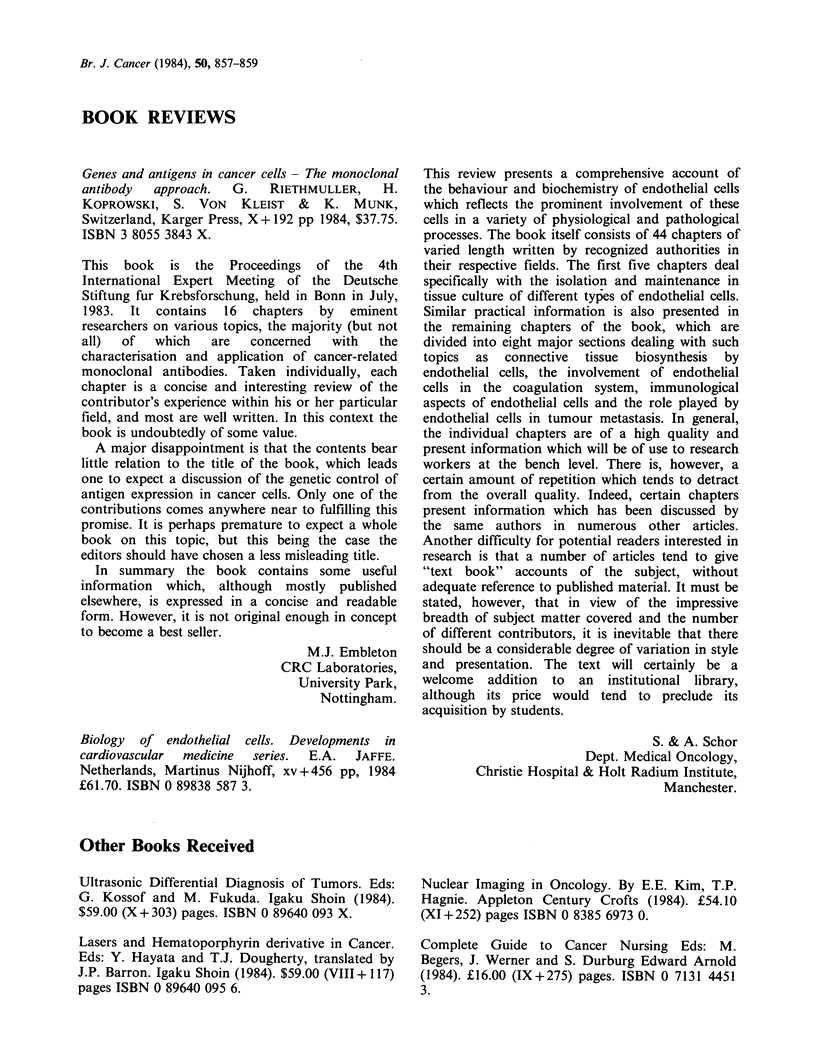# Genes and antigens in cancer cells - The monoclonal antibody approach

**Published:** 1984-12

**Authors:** M.J. Embleton


*Br. J. Cancer* (1984), **50**, 857–859

### BOOK REVIEWS

*Genes and antigens in cancer cells – The monoclonal antibody approach.* G. RIETHMULLER, H. KOPROWSKI, S. VON KLEIST & K. MUNK, Switzerland, Karger Press, X+192 pp 1984, $37.75. ISBN 3 8055 3843 X.

This book is the Proceedings of the 4th International Expert Meeting of the Deutsche Stiftung fur Krebsforschung, held in Bonn in July, 1983. It contains 16 chapters by eminent researchers on various topics, the majority (but not all) of which are concerned with the characterisation and application of cancer-related monoclonal antibodies. Taken individually, each chapter is a concise and interesting review of the contributor's experience within his or her particular field, and most are well written. In this context the book is undoubtedly of some value.

A major disappointment is that the contents bear little relation to the title of the book, which leads one to expect a discussion of the genetic control of antigen expression in cancer cells. Only one of the contributions comes anywhere near to fulfilling this promise. It is perhaps premature to expect a whole book on this topic, but this being the case the editors should have chosen a less misleading title.

In summary the book contains some useful information which, although mostly published elsewhere, is expressed in a concise and readable form. However, it is not original enough in concept to become a best seller.

M.J. Embleton
CRC Laboratories,
University Park,
Nottingham.